# Continuous roll-to-roll patterning of three-dimensional periodic nanostructures

**DOI:** 10.1038/s41378-020-0133-7

**Published:** 2020-04-20

**Authors:** I-Te Chen, Elizabeth Schappell, Xiaolong Zhang, Chih-Hao Chang

**Affiliations:** 1 Department of Mechanical and Aerospace Engineering, North Carolina State University, Raleigh, NC 27695 USA; 2 Walker Department of Mechanical Engineering, University of Texas at Austin, Austin, TX 78712 USA

**Keywords:** Nanoparticles, Optical materials and structures

## Abstract

In this work, we introduce a roll-to-roll system that can continuously print three-dimensional (3D) periodic nanostructures over large areas. This approach is based on Langmuir-Blodgett assembly of colloidal nanospheres, which diffract normal incident light to create a complex intensity pattern for near-field nanolithography. The geometry of the 3D nanostructure is defined by the Talbot effect and can be precisely designed by tuning the ratio of the nanosphere diameter to the exposure wavelength. Using this system, we have demonstrated patterning of 3D photonic crystals with a 500 nm period on a 50 × 200 mm^2^ flexible substrate, with a system throughput of 3 mm/s. The patterning yield is quantitatively analyzed by an automated electron beam inspection method, demonstrating long-term repeatability of an up to 88% yield over a 4-month period. The inspection method can also be employed to examine pattern uniformity, achieving an average yield of up to 78.6% over full substrate areas. The proposed patterning method is highly versatile and scalable as a nanomanufacturing platform and can find application in nanophotonics, nanoarchitected materials, and multifunctional nanostructures.

## Advanced Nanomanufacturing: Printing 3D nanostructures

A scalable printing process offers a high-yield way to produce 3D nanostructures. Repeating 3D nanostructures have useful properties such as controlling the transmittance and reflectance of light. They can also function as solar-cell or battery components and can form materials with novel properties. Chih-Hao Chang and his team from North Carolina State University, United States, developed a roll-to-roll patterning method to produce 3D nanostructures. Their system utilizes nanospheres that self-assemble into periodic patterns, followed by nanolithographic printing using laser light. In this technique, the diffraction of the laser light by the nanospheres creates 3D periodic nanostructures on an underlying photosensitive layer. Chang’s team demonstrated the process by producing crystals that more effectively reflect specific wavelengths of light. The team showed the process offers an assembly yield over 85%, without the need for expensive hardware.

## Introduction

Periodic three-dimensional (3D) nanostructures have been investigated in recent years according to their unique physical properties that only exist on the micro/nanoscale. For example, the distinct dispersion behavior of photonic crystals with periodic dielectric profiles can inhibit the propagation of photons with specific energy. Such a photonic energy bandgap can be used to control the transmittance and reflectance of light by designing the structure period^1–4^. The high surface-area-to-volume ratio of nanostructures can also be employed in the electrodes of fast-charging batteries^5^ and solar cells^6^. Furthermore, 3D nanostructures can overcome the physical limitations observed for the mechanics of macroscale materials, leading to mechanical metamaterials with novel properties. Recent studies have shown that periodic nanoarchitected or nanolattice materials have more favorable density scaling of their stiffness and strength than random porous microstructures^7^, can demonstrate larger recoverability^8–10^, and can exhibit uncommon behaviors such as negative Poisson’s ratio or stiffness^11–13^. Nanolattice materials can also exhibit interesting behavior in other physical domains, such as near-unity refractive index^14^, enhanced light trapping^15^, and low thermal conductivity^16^.

Given the advantages of periodic 3D nanostructures, a variety of “top-down” lithography processes have been pursued to demonstrate such structures. These include focused ion beam (FIB)^17^, two-photon polymerization^18^, and electron-beam lithography^19^, which can achieve precise patterning with the desired profile using a layer-by-layer approach. However, these techniques are serial processes that require point-by-point scanning and have limited patterning throughput. They can be time-consuming and prohibitively expensive as the sample size increases. On the other hand, nanolithography approaches that are parallel in nature have also been investigated. One example is laser interference lithography (IL), in which the interference of mutually coherent laser beams can create periodic 3D intensity patterns over large areas^20–24^. These techniques are also highly versatile, and the lattice geometry can be tailored by controlling the number of beams and their respective incident angles^25^. However, these systems require extensive optical elements, precision alignment stages, and a coherent light source. Therefore, such approaches are suitable for a research environment but can be limited in a manufacturing setting. Other lithography configurations based on light interference employ a lithographically defined diffractive mask^26,27^ or a refractive prism^28^. In these methods, the optical elements are illuminated to create multiple off-axis propagating beams that can interfere and create a 3D intensity pattern. However, the pattern relies on high-quality phase masks or precisely cut prisms, which require customization and are expensive to make.

Another nanofabrication approach relies on the “bottom-up” self-assembly of colloidal elements to build 2D or 3D structures^29–35^. For the assembly of a single layer or few layers on a planar surface^32,34,36–42^, these colloids can be used as physical masks for deposition^43,44^ or etching^45–47^ processes. For 3D assembly, these methods can be employed to fabricate opal or inverse opaline structures^5,32^, hemispherical metal shells^48^, or hierarchical nanopore arrays^5,45,49,50^. Recent efforts have also demonstrated large-area colloidal crystals using continuous roll-to-roll systems^51,52^. However, the self-assembly of colloidal crystals can only yield nanostructures with limited lattice geometry, such as opal/inverse opal or hexagonal close-packed arrays. The resulting assemblies are also typically qualitatively studied through visual observation or frequency analysis of the spectra^35–41,51^, which provides limited information regarding the average lattice spacing and relative assembly order. Therefore, precise quantitative metrology of the assembly yield rate over a large area is challenging. In recent work, various groups have demonstrated that colloidal nanospheres can be used as a near-field optical element for nanolithography to create 3D periodic nanostructures^53–58^, nanovolcanoes^59,60^, and hierarchical structures^61^. However, in these proof-of-concept demonstrations, the colloidal assembly and lithography were performed manually within a limited area. The ability to perform high-throughput 3D printing of nanostructures is a critical step towards nanomanufacturing.

In this work, we introduce a roll-to-roll system for continuous patterning of 3D periodic nanostructures using light diffraction from self-assembled colloidal nanospheres. This system encompasses both “bottom-up” and “top-down” approaches, namely, automated Langmuir-Blodgett assembly for continuous self-assembly of colloidal nanospheres and near-field lithography for direct 3D patterning. In this manner, nanospheres are assembled into an ordered array and illuminated to create a 3D intensity distribution for patterning in a streamlined process. By controlling the size of the sphere and exposure wavelength, the light interactions with the nanosphere array can be modeled to design different lattice geometries. This continuous process is highly scalable, where the maximum area is limited only by the width of the liquid trough, and has been used to demonstrate uniform patterning on 2 × 8 in^2^ flexible substrates. The pattern precision of this system has been quantitatively characterized by analyzing scanning electron microscope (SEM) images to study the influence of system parameters, the long-term repeatability, and the pattern uniformity. The proposed system enables continuous printing of 3D periodic nanostructures and can be a versatile and scalable nanomanufacturing platform.

## Experimental approach

**Fig. 1:**
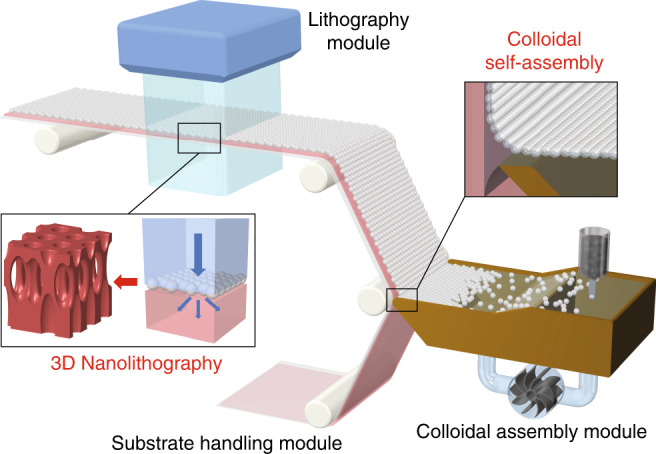
Schematic of the roll-to-roll colloidal 3D nanolithography system. The system consists of subsystem modules for colloidal assembly, substrate handling, and 3D nanolithography

The proposed roll-to-roll 3D nanostructure patterning system is illustrated in Fig. 1. The whole system is the integration of three subsystems, which include substrate handling, colloidal assembly, and lithography modules. The substrate handling module consists of a roll-to-roll system that can transport advancing substrates with constant velocity and coating rate. The colloidal assembly module consists of a Langmuir-Blodgett trough, where colloidal nanospheres self-assemble into a monolayer of a hexagonal close-packed (HCP) lattice on the surface of a moving fluid. The nanospheres are immersed in a butanol suspension, which is introduced on a water surface using a motorized syringe pump with a constant injection rate. The butanol then evaporates, with the nanospheres assembling into periodic patterns at the air–water interface. The nanosphere array monolayer can then be transferred to a substrate through the substrate handling module, forming a 2D colloidal crystal upon drying.

The HCP nanosphere array monolayer is then employed as the phase element for near-field nanolithography. This process occurs in a continuous fashion while the substrate handling module conveys the substrate to the lithography module. Here, a laser diode is used as the light source, which consists of a diode mount system with a lens set to collimate and expand the laser beam (more details of the optical setup can be found in Supplementary Section A). Under normal illumination, the nanosphere array diffracts the light into discrete propagating orders, creating a 3D intensity distribution in the near field. This phenomenon is also known as the Talbot effect, which is then recorded by the underlying photoresist layer in close proximity to the spheres. Note that lithographic exposure is performed while the substrate is scanned with constant velocity, which provides continuous exposure of the photoresist at a constant dose. After the lithography process, the assembled nanospheres can be removed by sonication, and the developed photoresist forms 3D periodic nanostructures. Using this process, photoresist can be continuously patterned with 3D periodic nanostructures in a roll-to-roll fashion.

## Results and discussion

**Fig. 2:**
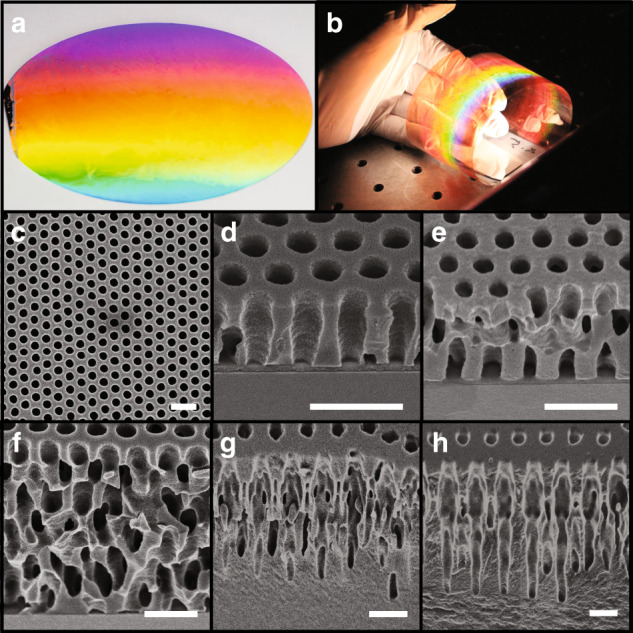
Fabrication results of the roll-to-roll colloidal 3D nanolithography system. **a** One micron silica nanospheres assembled on a 100-mm Si substrate. **b** Diffraction from periodic 3D nanostructures with a 500-nm period patterned on a 50 × 200-mm^2^ PET film. **c** Top-view SEM image of photoresist nanostructures fabricated using 500 nm silica nanospheres and 325 nm exposure. Cross-section SEM images of the nanostructures fabricated using 500 nm silica nanospheres and exposure to a **d** 325-nm laser, **e** 405 nm laser diode, and **f** 450 nm laser diode. **g** Cross-sectional SEM images of the nanostructure fabricated using 1 μm silica nanospheres and a 325-nm laser. **h** Cross-sectional SEM image of the nanostructure fabricated using 500 nm silica nanospheres and a 405-nm laser diode

The fabrication results of the R2R 3D patterning tool are shown in Fig. 2. The sample prior to the lithography process is shown in Fig. 2a, where a 100-mm substrate with 1-μm-thick photoresist is coated with a monolayer of silica nanospheres with a 1000-nm diameter. The substrate is illuminated by a broadband halogen lamp, generating a rainbow appearance from the first-order diffraction of the periodic nanospheres. The assembled nanospheres can then be exposed to create 3D nanostructures, as illustrated in Fig. 2b. Here, 3D nanostructures with a 500-nm period were patterned using a laser diode with a 405-nm wavelength on a 50 × 200-mm^2^ PET film. In this sample, the nanospheres were removed before development, and the rainbow appearance is induced by the diffraction of the 3D photoresist structures. Note that the photoresist structure can act as a sacrificial template for subsequent material deposition processes such as physical vapor^34,36^ or atomic layer deposition, which can yield 3D nanostructures in oxides or metals^10,14,15^.

### Effect of *γ* on the Talbot distance

Different nanostructures patterned using this method are shown in Fig. 2c–h. Figure 2c depicts a top-view SEM image of the fabricated structures patterned using a 500-nm silica nanosphere array exposed to a 325-nm HeCd laser. Here, the hole array marks the locations where the spheres were located, which created a high-intensity focus. Figure 2d–f demonstrates the nanostructures patterned using 500 nm silica nanospheres but different light sources with wavelengths of 325, 405, and 450 nm, respectively. The structures shown in Fig. 2g, h were patterned using 1 μm diameter silica nanospheres exposed to 325 and 405 nm wavelength illumination, respectively. It can be observed that the lattice period and unit-cell geometry for each sample are different, with varying degrees of structural complexity. The lithography results illustrate the ability of the proposed approach to build desired architectures by controlling the nanosphere diameter and incident wavelength.

The 3D pattern formation mechanism can be attributed to the Talbot effect^62,63^, which is induced by the interference of light diffracted from the periodic nanosphere array under normal illumination^53,54^. The near-field intensity pattern is then recorded by the underlying photoresist, creating 3D periodic nanostructures, which can be controlled using different nanosphere sizes or exposure conditions. The Talbot distance (*z*_*t*_), or the axial period along the optical axis, is well studied and depends on the incident wavelength and period of the diffractive element^62,63^. The normalized Talbot distance is given by

1 $$\frac{{z_t}}{\Lambda } = \frac{\gamma }{{1 - \sqrt {1 - \gamma ^2} }}$$

where *λ* is the wavelength of the illumination, *n* is the refractive index of the medium, Λ is the lateral period of the periodic element, and *γ* is a unitless parameter defined as *γ* = *λ*/*n*Λ. The period can be calculated as $\Lambda = D\sqrt 3 /2$ for a hexagonal lattice with nanosphere diameter *D*^51^. This analytical relationship determines that the lattice periods in the lateral and axial directions, namely, Λ and *z*_*t*_, respectively, can be independently controlled by tuning the ratio of the sphere diameter to incident wavelength.

**Fig. 3:**
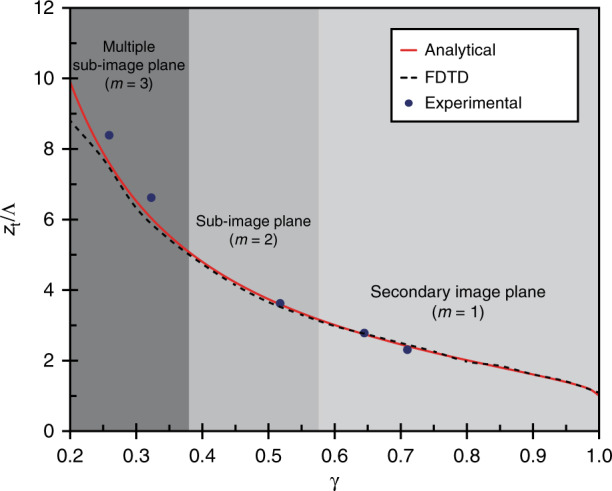
Comparision of simulation model and experimental results. Normalized Talbot distance versus *γ* parameter for the analytical model, FDTD simulation, and experimental data

This model can be validated by the fabrication results, and Fig. 3 depicts the normalized *z*_*t*_ vs *γ* parameter results for the analytical model and experimental data. The results of a numerical model using the finite-different time-domain (FDTD) method are also plotted for comparison. The fabricated structures are shown in Fig. 2d–h, which have measured *z*_*t*_ of 1.6 µm, 1.2 µm, 1.0 µm, 6.6 µm, and 5.2 µm for *γ* parameters of 0.52, 0.65, 0.71, 0.26, and 0.32, respectively. The effect of designing the *γ* parameter to control *z*_*t*_ is evident in Fig. 2d–f, where 500 nm silica nanospheres are illuminated by different light sources. As the wavelength of the light source increases from 325 to 405 and 450 nm, *γ* increases from 0.52 to 0.71, resulting in a smaller *z*_*t*_. This model presents a precise method to control the periods of the patterned 3D nanostructures using the normalized *γ* parameter.

Beyond the control of *z*_*t*_, the unit-cell geometry of the structures is also relevant to *γ*. This can be attributed to the fact that the angles of the light diffracted from the nanosphere array are proportional to *γ*. A larger *γ* value generally yields simpler periodic structures because only the 1st–order diffraction is the propagating modes in the photoresist^54^. In previous work, we outlined that the cutoff *γ* parameters of $1/\sqrt 3$ and $1/\sqrt 7$ for 2^nd^- and higher-order diffractions exist^53,54^, respectively, as denoted by the boundaries in Fig. 3. This can be observed in the fabricated structures depicted in Fig. 2d, f, where *γ* is 0.52 and 0.71, respectively. Here, the unit-cell geometry can be observed to be relatively simple since the intensity pattern consists of only the primary Talbot and phase-reversed images. Figure 2g, h illustrates the increase in the structure complexity change with lower *γ*. Here, larger nanospheres are used in both cases with different wavelength light, yielding *γ* parameters of 0.26 and 0.32, respectively. In both cases, $\gamma \,<\, 1/\sqrt 7$, so the 3^rd^–order diffraction is included, which results in unit-cell geometries that are more complex than those shown in Fig. 2d–f.

### Characterization of the assembly yield

The quality of the nanosphere assembly is a critical factor for the patterning processes; therefore, it is important to quantitatively characterize the assembly yield of the nanosphere array. Since the Talbot effect is based on the illumination of a periodic element with high spatial-phase coherence, any assembly defects can lead to random light scattering and poor exposure. This is especially critical since self-assembly systems, such as colloidal nanospheres, are prone to assembly defects. To demonstrate the proposed system as a potential nanomanufacturing tool, the assembly yield, long-term repeatability, and large-area uniformity must be considered. Once these parameters have been examined, the effect of lithography will be examined to establish the overall system patterning yield.

Colloidal assembly at an air–liquid interface depends on a number of system and environmental conditions. On the one hand, deposition parameters, such as the coating speed, suspension concentration, and carrier fluid flow rate, directly influence the self-assembly conditions. On the other hand, environmental disturbances, such as vibration and carrier fluid perturbation, also contribute to assembly defects. One of the most important parameters is the pairing of the coating rate and the injection rate, which controls the equilibrium between the number of input and output nanospheres. Insufficient injection can lead to discontinuity in the assembly, resulting in a high number of vacancies and a low nanosphere packing density. However, excessive nanosphere injection results in a multilayer assembly that can form cracks after transfer to a substrate^32,34^.

**Fig. 4:**
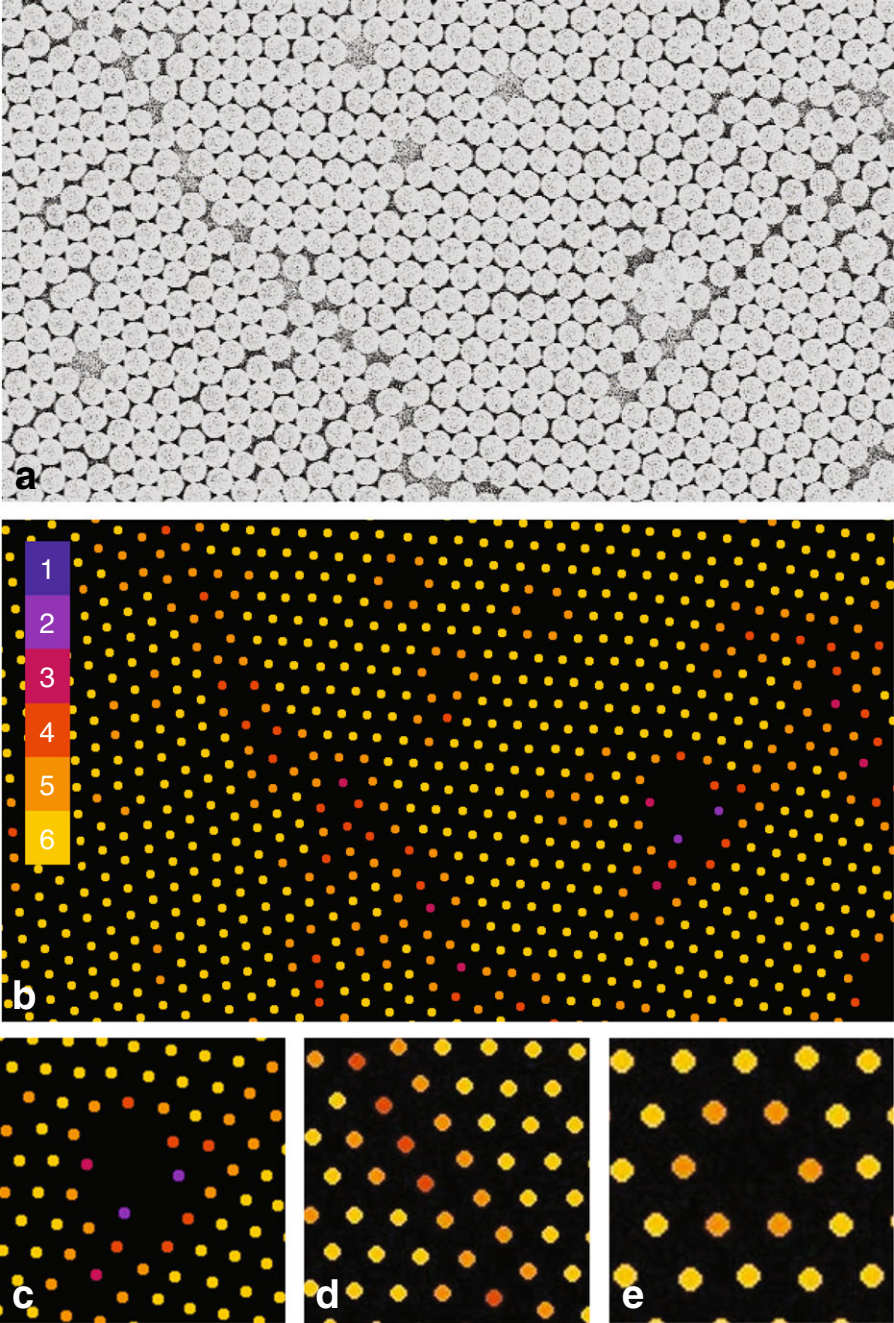
Defect inspection process using SEM imaging. **a** Original top-view SEM image with **b** the connection mode for each sphere identified. Assembly defects, such as **c** aggregation, **d** grain boundary dislocation, and **e** a point vacancy, can be identified

Most existing works on yield inspection of colloidal assemblies rely on observation using top-view optical microscopy or SEM imaging. Therefore, the analysis is typically limited to qualitative measures, such as the number of assembly layers (monolayer, bilayer, multilayers, etc.) and continuity of the assembled film. These techniques are insufficient for quantifying the spatial-phase coherence required for near-field lithography purposes. For a more quantitative analysis of the assembly and lithography yields, we introduce an inspection algorithm using electron microscopy. Implemented using ImageJ, this approach is based on precisely analyzing the number of nearest neighbors for each nanosphere. This parameter can be used to quantify the crystallinity of the nanosphere assembly and the defect modes. A schematic diagram of the algorithm is shown in Supplementary Section B. The operation of this algorithm is illustrated in Fig. 4. Initially, a top-view SEM image of the nanosphere assembly is recorded, as shown for the array of 500 nm diameter silica spheres in Fig. 4a. The center of every individual sphere is then located by finding the centroid of the intensity profile. For each sphere, the algorithm then calculates the number of nearest neighbor spheres that are at a distance of the sphere diameter. This process determines the number of neighboring spheres that are in direct contact, with a range from six for the crystalline HCP lattice to zero for a single isolated sphere. This analysis results in an assembly packing contour, as depicted in Fig. 4b, which plots the number of neighbors for each sphere using different colors for different numbers of neighbors. By observing the contour, the number of defect modes can be identified. These include aggregation of nanospheres, grain dislocation, and point vacancies, as shown in Fig. 4c–e, respectively.

The electron microscopy inspection method can calculate the packing statistics to determine the assembly yield. Here, the yield can be defined as the percentage of spheres that are in the HCP lattice without restriction to any particular angular orientation. First and foremost, spheres with six neighbors are classified as a single HCP grain. However, spheres with 5 or even 4 neighbors can represent the boundaries of a single crystal grain. This can be observed in Fig. 4d, e, where two common cases of spheres with five neighbors are a dislocation and a point vacancy, which can be considered as a part of an HCP grain. Most spheres with high defective assembly states, such as loose packing or particle aggregation, have less than four neighbors, as shown in Fig. 4c. Therefore, two yield parameters are defined in this work, *η*_5_ and *η*_6_, corresponding to the percentages of nanospheres with five or six neighbors and only those with six neighbors, respectively. Note that the ratio of these two yields also gives a rough prediction of the average size of each single grain.

**Fig. 5:**
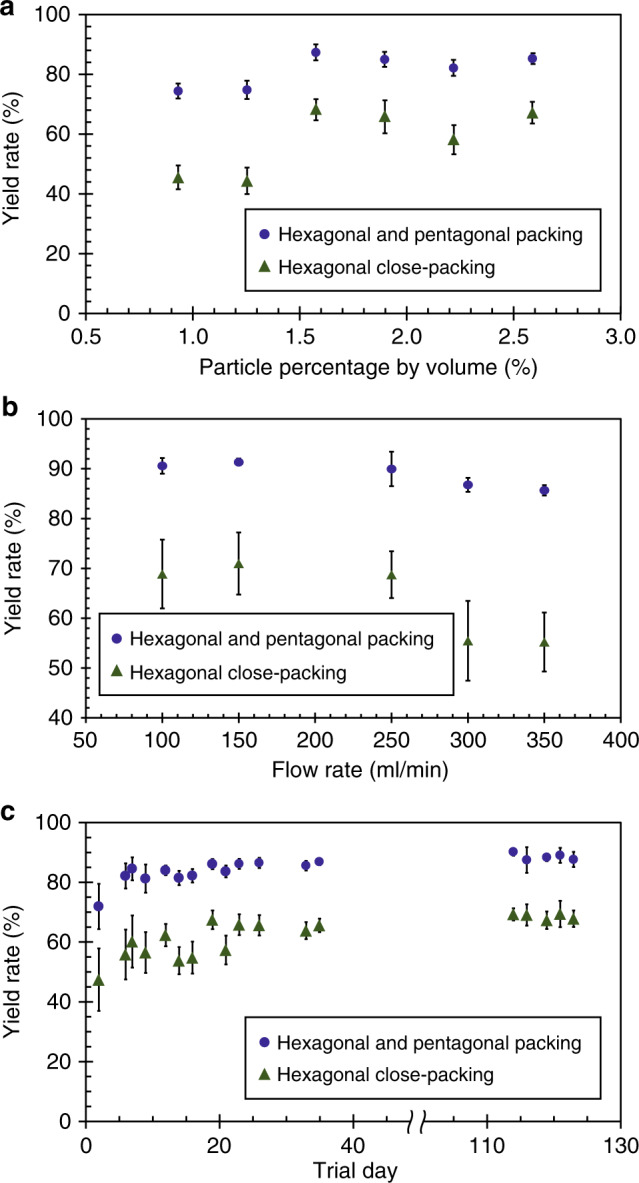
Yield analysis of the assembly process. Measured yield rate for different **a** suspension concentrations and **b** carrier fluid flow rates. **c** Long-term repeatability of the yield rate

Using the inspection algorithm, the parameters of the R2R assembly process can be examined to quantify the assembly yield, as shown in Fig. 5. Here, the assembly yield is plotted versus the nanosphere concentration, carrier flow rate, and trial date, and the error bars are the standard deviation of the yield based on 54 to 175 measurements. The first important parameter is the nanosphere concentration in the suspension, and its effect on the assembly yield is depicted in Fig. 5a. In this experiment, the suspension volume fraction is adjusted from 0.9 to 2.6%, while the other system parameters remain the same. The result shows that the average *η*_5_ is 79.5% when the concentration is below 1.6% and improves to 87.2% when the concentration is above 1.6%. This indicates that keeping the number of nanospheres constant cannot guarantee the assembly quality. A low packing density can be observed in the SEM images for concentrations below 1.2% (as shown in Supplementary Section C), resulting in more vacancies and smaller HCP grains. On the other hand, a higher concentration colloidal suspension tends to form aggregates and requires a longer sonication time prior to assembly. In this work, the suspension with a 1.6% concentration by volume has the highest assembly yield and is used in all subsequent experiments.

The carrier fluid flow rate also plays a key role in the assembly yield because a higher flow rate creates a larger relative velocity between the fluid and colloidal film, providing a larger force for packing the assembly. However, a high flow rate can degrade the laminar flow condition and lead to ripples on the surfaces, which deteriorates the assembly. The contribution of the carrier fluid flow rate to the assembly yield is plotted in Fig. 5b. Here, *η*_5_ is ~90% and consistent over different flow rates, whereas *η*_6_ drops from 70 to 55% when the flow rate is higher than 300 ml/min. This result illustrates that the ripples along with the high flow rate disturb the nanosphere monolayer and reduce the assembly yield. This flow rate determines the overall system assembly throughput, defined by the nanosphere coating rate of 3 mm/sec. A higher throughput of up to 1 cm/sec can potentially be achieved by designing proper width and depth dimensions of the Langmuir-Blodgett trough to maintain a fully developed laminar flow at a higher fluid flow rate.

To demonstrate the long-term repeatability of the R2R assembly system, the assembly yield is examined over a 120-day period, as shown in Fig. 5c. In these experiments, 100 mm silicon substrates were fully coated with 1 µm silica nanospheres and analyzed using the SEM inspection algorithm. For each sample, 25 images forming a 5 × 5 matrix at the center of the substrate were taken for image analysis. Here, *η*_6_ and *η*_5_ range from 48.0 to 70.0 and 72.5 to 90.7%, respectively, and both gradually improve over time. For the first 20 days, the standard deviations of *η*_6_ and *η*_5_ are 6.0 and 3.5% but improve to 3.6 and 1.9% afterward, respectively. This trend shows that the assembly yield continuously improves as the user becomes more familiar with the system. The R2R assembly system is located in a room where the temperature and humidity can vary from 65 °F to 75 °F and 30 to 60%, respectively, and better environmental control can potentially further improve the assembly yield.

**Fig. 6:**
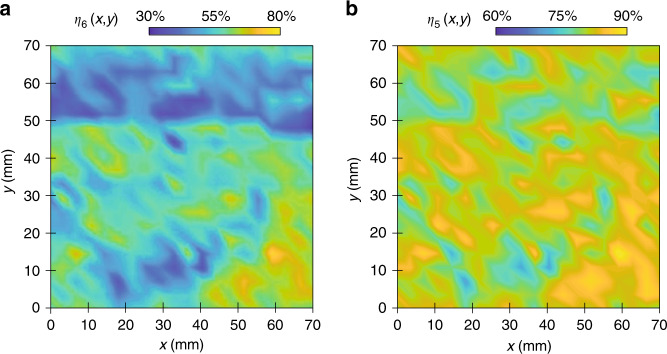
Measured yield of the colloidal assembly process. Assembly yield contours over a 70 × 70 mm^2^ area for the **a** *η*_6_ and **b** *η*_5_ yield rates. The assembly scanning direction is along the *x*-axis, where translational symmetry can be observed

To demonstrate the capability of the proposed system as a manufacturing tool, it is important to quantify the assembly yield uniformity over a large area. This is achieved by using automated SEM imaging of fully patterned substrates and analysis with the inspection algorithm. From these data, the 2D yield contours *η*_6_(*x*, *y*) and *η*_5_(*x*, *y*) can be plotted, as shown in Fig. 6. Here, the contours are constructed from 400 SEM images forming a 20 × 20 matrix over a sampling area of 70 × 70 mm^2^. The 100 mm silicon substrate is coated with 1000 nm silica nanospheres, and representative SEM images of the contours are presented in Supplementary Section D. The yield contour of *η*_6_ demonstrates an average yield of 54.7% with a standard deviation of 8.2%. Different regions can be identified, where the maximum yield of 75.1% is achieved towards the lower right corner (*x* = 60 mm, *y* = 20 mm). This corresponds roughly to the center of the substrate. Of particular interest are the two green–blue boundaries parallel to the coating orientation at approximately vertical positions of 15 mm and 50 mm, where *η*_6_ is ~30%. The SEM images of these two areas show that the assembly contains a high density of defects, which significantly reduces the assembly yield. This can be attributed to the lateral fluid velocity gradients at the edge of the trough and the nonuniform coating due to the circular shape of the substrate. Spatial variation of the coating front can result in uneven transfer of the monolayer, which increases nanosphere grain dislocation and multilayer formation at the air-liquid interface. The yield contour of *η*_5_ is better, with an average yield of 81.1% and a standard deviation of 3.1%, but a similar spatial distribution of defects can be observed.

The yield contours also reveal the directional effect of the nanosphere assembly process, which shows less variation along the coating direction. The data highlight this phenomenon, where the standard deviations of *η*_6_ parallel and perpendicular to the coating direction are 6.1 and 8%, respectively. This can be attributed to the translational symmetry of the coating process, where the assembly conditions do not change along the scanning direction during steady-state operation. However, it is important to note that when a defect is formed on the substrate, the local disorder can disturb the subsequent packing and expand the defect along the coating direction, which can be observed in the macroscale streaks on the sample. This result also indicates that the assembly yield is more sensitive along the perpendicular direction due to the edge effects and flow nonuniformity imposed by the LB trough. Therefore, the width and shape of the trough can be further designed to mitigate the edge effects. Other important parameters, such as the temperature, humidity, and pulling angle during the assembly process, can also affect the assembly quality and will be studied in future work.

### Characterization of the lithography yield

Beyond the assembly process, the SEM inspection algorithm can also be used to analyze the lithography process for patterned 3D nanostructures. Because the Talbot effect is closely related to the period of the phase element, only the regions under an HCP assembly can result in a 3D intensity pattern. For regions with significant assembly defects, scattered light can reduce the pattern fidelity and lead to photoresist collapse. It is therefore important to examine how the nanosphere assembly yield translates to the 3D lithography yield. For positive photoresist, the positions below the spheres upon normal illumination will be holes due to the near-field focusing effect^55^. Therefore, the exposure dose can be estimated by measuring the hole diameter using top-view SEM imaging. In the case of underexposed photoresist, the hole diameter will be relatively small, and vice versa. On the other hand, a region that is overexposed will show structure collapses, which can significantly degrade the patterning yield. More details regarding the study of the hole size and the exposure yield are shown in Supplementary Section E.

**Fig. 7:**
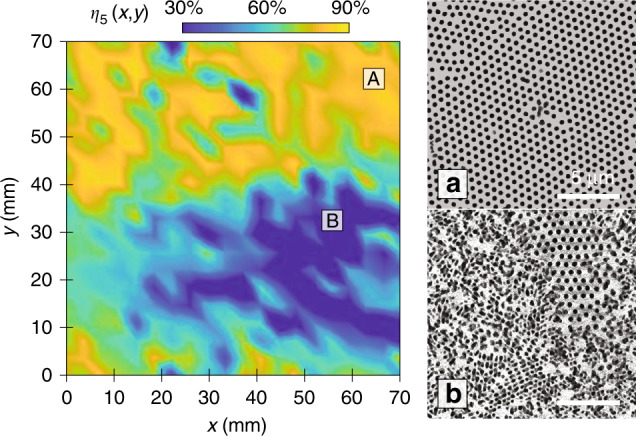
Lithography yield for static exposure. Measured yield contour *η*_5_ over a 100-mm substrate. Top-view SEM images of regions with **a** a high yield, denoted by location “A,” and **b** an overexposed structure, denoted by location “B”

To examine the effect of exposure, two illumination methods have been examined for the lithography process. The baseline is static exposure, where the substrate is held stationary and exposed to the far-field illumination of a point source. This will be compared with scanning exposure in the roll-to-roll system, where the substrate is scanned under a collimated beam with a Gaussian profile. The nominal exposure dose is 110 mJ/cm^2^ for the static exposure using a HeCd laser with a 325-nm wavelength. The patterning yield contour for *η*_5_ under the static exposure condition for a 100-mm silicon substrate is shown in Fig. 7. The contour is constructed from an analysis of 400 SEM images over a 70 × 70 mm^2^ area. Here, it can be observed that the yield varies greatly across the area, reaching a maximum of 86.6% at location “A” and a minimum of 30.2% at location “B.” The corresponding top-view SEM images of these two locations are depicted in Fig. 7a, b, respectively. The yield has a standard deviation of 13.7% across the whole substrate, significantly higher than those observed for the assembly yield. This can be attributed to the 2D intensity distribution of the point source, resulting in different exposure doses across the substrate. The lowest yield is observed at and around location “B,” which coincides with the center of the beam with the highest intensity. The SEM image for this location illustrates that the photoresist has been overexposed, resulting in structure cracking and collapse due to mechanical instability. The dose is lower in the outer ring away from the center at point “A,” where the structures have a proper exposure dose and are intact. The average patterning yield rate *η*_5_ around location “A” is 82.7% and is similar to the average assembly yield rate prior to the lithography process.

**Fig. 8:**
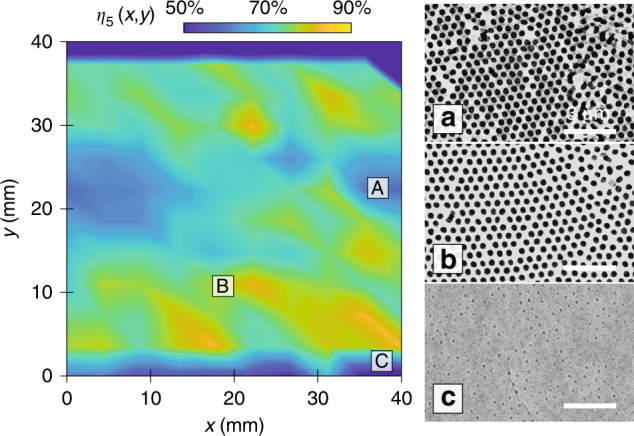
Lithography yield for scanning exposure. Measured yield contour *η*_5_ over a 100-mm substrate. Top-view SEM images of regions with **a** an overexposed structure, denoted by location “A,” **b** a high yield, denoted by location “B,” and **c** an underexposed structure, denoted by location “C”

Better exposure results were obtained for the scanning exposure with a 50-mm diameter light source expanded from a laser diode using the roll-to-roll system. The light source is calibrated by measuring the intensity distribution over the illumination area of the collimated beam. The nominal exposure dose is 40 mJ/cm^2^ for the scanning exposure, which can be controlled based on the integral of the intensity with the scanning time, as described in further detail in Supplementary Section E. The patterning yield contour for *η*_5_ under the scanning exposure condition is illustrated in Fig. 8. Here, 120 SEM images over a 40 × 40 mm^2^ area are analyzed to generate the contour. The patterning yield shows bilateral symmetry along the scanning direction with a clear centerline, which can be attributed to the Gaussian dose profile shown in Supplementary Section E. It is interesting to note that the maximum yield is not located along the centerline but in regions offset towards the top and bottom. In the central region within 8 mm from the centerline labeled “A”, the exposure dose is approximately 52 mJ/cm^2^, slightly above the nominal exposure dose of 42 mJ/cm^2^. Figure 8a depicts the structures in this region, where some unclear features from overexposure can be observed that reduce the patterning yield to 67.9%. Figure 8b depicts the structures in location “B,” which is 8–18 mm from the centerline, and the dose varies from 25 to 42 mJ/cm^2^. The contour illustrates that this is the most suitable exposure condition, reaching a maximum yield of 82.6% with an estimated dose of ~40 mJ/cm^2^. The lowest patterning yield is below 50% and is found in region “C,” where the dose is below 20 mJ/cm^2^. The dose here is insufficient to fully expose the photoresist, resulting in partially exposed shallow spots, as shown in Fig. 8c.

It is important to note that while the yield contours greatly differ between the static and scanning exposure configurations, the peak yield values are similar. The yield rates of the nanosphere assembly and 3D static and scanning exposure processes are summarized in Table 1. The statistical data illustrate that the average yield rate, *η*_5_, slightly decreases from 81.1 to 78.6% and 74.8%, and the standard deviation increases from 3.1 to 6.3 and 3.4%. This result indicates that after the lithography process, the structure yield and uniformity can further degrade due to over- or underexposure. This also implies that given the correct exposure dose, the lithography process does not further degrade the nanosphere assembly yield. Therefore, the assembly yield represents the key factor limiting the overall patterning yield of this process. It is important to note that the yield uniformity can be improved by reducing the variations in the exposure profile. Currently, the result shows that the scanning exposure configuration can achieve a yield deviation of 4.1% along the scanning direction but leads to a much worse deviation (~20%) in the perpendicular direction. A feasible solution to overcome this directional effect is 2D raster scanning with integral multiple 1D scanning at fixed intervals, where the Gaussian beam profile can be smoothed in both directions. An extended light source consisting of cylindrical lenses or multiple laser diodes can also be used, which will broaden and smoothen the intensity profile in the perpendicular direction. The resulting illumination will not only be more uniform but also be able to pattern larger areas.

**Table 1:** Yield rate comparison before and after lithography processes

Process	*η*_6_ Average (%)	*η*_6_ Std. dev. (%)	*η*_5_ Average (%)	*η*_5_ Std. dev. (%)
Before lithography	54.7	8.2	81.1	3.1
Static lithography	55.2	11.8	78.5	6.3
Scanning lithography	34.9	6.4	74.8	3.4

Another potential research direction is in situ measurement of the colloidal assembly quality during coating using optical techniques. The SEM image analysis proposed in this work relies on postprocess inspection and does not allow for real-time correction of the assembly parameters. Potential techniques include scatterometry, with which optical properties such as the diffraction spectra or thin-film interference of the nanosphere monolayer at the air–liquid interface can be analyzed to determine the packing density and order. Such information can allow real-time feedback to correct for assembly defect modes. Moreover, the current inspection algorithm is based on top-view SEM imaging, and the fidelity of the structure below the surface cannot be determined. By applying the optical diffraction characteristics, further insights into the 3D nanostructure can be obtained.

Using the proposed approach, various nanospheres from 100 nm to 2 µm in diameter and illumination with wavelengths from 325 nm to 450 nm have been successfully employed. These nanosphere diameters and illumination configurations, which correspond to a wide range of *γ*, can be used to pattern a diverse range of periodic nanostructures for application in nanophotonics, microfluidic systems, and nanoarchitected materials. Moreover, this work also demonstrates patterning on a curved PET film, which can be employed to build 3D periodic nanostructures on flexible or nonplanar substrates for wearable photonics.

## Conclusion

In this work, we investigate a roll-to-roll nanolithography system capable of continuous patterning of 3D nanostructures on diverse substrates. By harnessing the near-field optical effects of self-assembled colloidal nanospheres, this method is scalable for large-area patterning. This approach is also versatile, and different unit-cell geometries can be designed by varying the wavelength relative to the nanosphere diameter. The temporal and spatial precision of this system have been quantitatively analyzed using SEM imaging and the inspection algorithm. The long-term repeatability demonstrates an assembly yield over 85%, and the standard deviation is <2%. The yield uniformity was also examined over the whole-substrate area, with the results showing a directional effect along the assembly direction that can be attributed to the finite width of the LB trough. The yield contour for the scanning exposure condition also shows that the lithography process does not significantly degrade the assembly yield, and the patterning uniformity can be further improved by scanning the laser beam such that multiple Gaussian profiles overlap. Without expensive hardware and with the pattern area limited by the trough width, this system is a scalable platform for continuous printing of periodic 3D nanostructures.

## Materials and methods

### Substrate preparation

In this work, silicon substrates (100 mm single-side polished Si wafer, University Wafer) and microscope glass slides (75 × 25 × 1.2 mm^3^ plain glass, VWR) were used. Flexible or stretchable substrates such as polyethylene terephthalate (PET) films (454 Melinex PET, Tekra) or polydimethylsiloxane (PDMS Sylgard 184, Dow Corning Corp.) were also implemented. An antireflection layer (ARC i-con-7, Brewer Science) with a thickness of ~100 nm was used to reduce back reflection for silicon substrates. Different thicknesses of photoresist (PFi-88A2, Sumitomo) were then spin-coated or wire-bar coated to thicknesses of 1.5–3 µm.

### Colloidal assembly

The colloidal suspension used consisted of silica nanospheres (silica spheres, Fiber Optic Center Inc.) and butanol (1-butanol, Sigma-Aldrich) at various concentrations. During initial mixing, the suspensions were sonicated (5510 ultrasonic cleaning system, Branson) for 4 h in advance to disperse the nanospheres and mitigate aggregation. Further sonication was necessary if aggregates were found after long shelf storage. Nanospheres from 100 nm to 2 μm in diameter with concentrations from 0.9 to 2.7% by volume were used in this work. The colloidal assembly module was based on a Langmuir-Blodgett trough with constant flow (VFX-100, Versuflex). The injection rate was from 0.08 to 0.3 ml/min, corresponding to deposition speeds from 1.5 to 3 mm/s for different colloidal suspensions. A carrier fluid flow rate range of 100–350 ml/min was feasible in this study to form nanosphere arrays with different degrees of assembly yield.

### Lithography exposure

The exposure dose for the lithography process was varied depending on the photoresist sensitivity to the corresponding light source. In this work, the suitable exposure doses ranged from 105 to 125 mJ/cm^2^ for a 325-nm HeCd laser, 40–42 mJ/cm^2^ for a 405-nm laser diode, and 140–160 mJ/cm^2^ for a 450-nm laser diode. After removal of the nanospheres using a sonication bath, the exposed stack was immersed into the developer solution (CD-26, MicroChem Corp.) for 2 min. A subsequent deionized water cleaning step for 1 min was employed to remove the residual developer.

## Supplementary information

Revised supplementary information

graphical abstract
