# Supplementary material for: Continuous roll-to-roll patterning of three-dimensional periodic nanostructures
Source: Microsyst Nanoeng. 2020 Apr 20;6:22. doi: 10.1038/s41378-020-0133-7 (PMC8433208; doi:10.1038/s41378-020-0133-7)
Supplement: Supplementary file 1 — Revised supplementary information [file 41378_2020_133_MOESM1_ESM.pdf]

Continuous Roll-to-Roll Patterning of Three-Dimensional Periodic Nanostructures

*I-Te Chen, Elizabeth Schappell, Xiaolong Zhang, Chih-Hao Chang**

Department of Mechanical and Aerospace Engineering, North Carolina State University, Raleigh, NC 27695, USA

*Corresponding author: chichang@ncsu.edu

Supplementary Section A: Lithography Module

The lithography module consists of a laser diode as the light source and a lens set as the expander. The overall setup is shown as below.

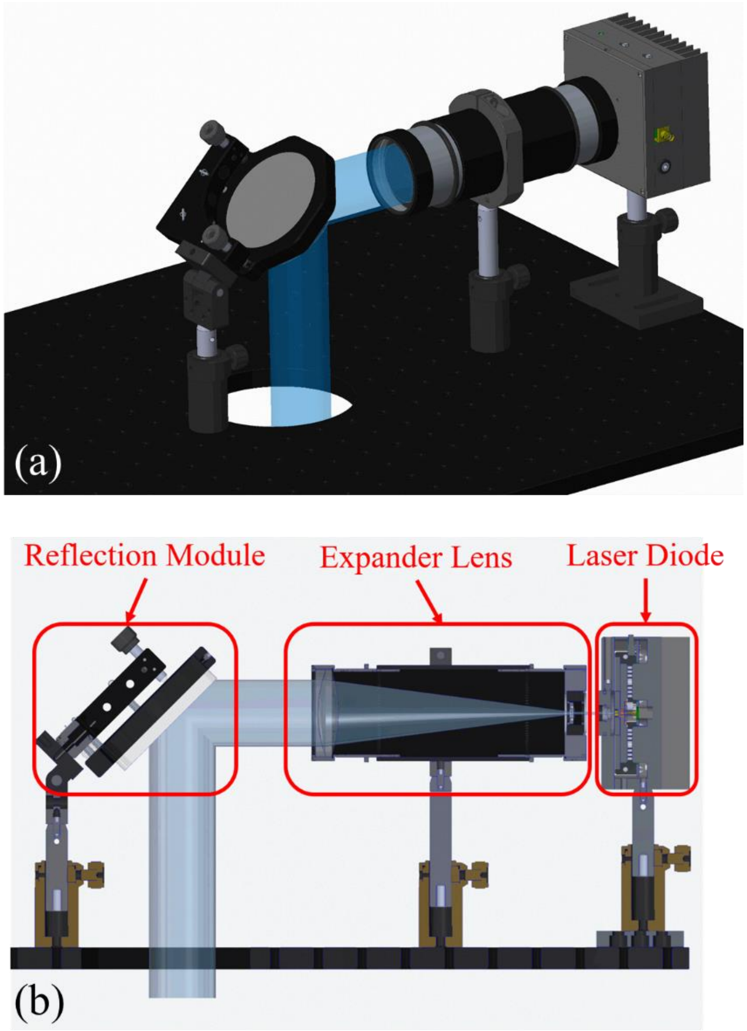

Figure S1 Schematic of the lithography module. (a) Overview of the module. (b) The cross-section view of the module.

The light source used in this work is a 405 nm UV laser diode (DL5146-101S, 40 mW, Thorlabs) and a 450 nm blue laser diode (PL TB450B, 1.6 W, Osram) fixed on a laser diode mount (LTC100-A, Thorlabs). The laser beam size from the diode is expanded by 24 times using a lens set which pairs a concave lens (LD2746-A, f = -6.0 mm, Thorlabs)

with a convex lens (AC508-150-A, f = 150.0 mm, Thorlabs). The expanded plane wave is then reflected by a mirror onto the sample with colloidal nanosphere monolayer for lithography. The intensity profile is estimated by analyzing the brightness distribution of the illumination pattern on a screen, which can then be used to calculate the intensity integral along the scanning direction to get the transverse intensity distribution as shown in Fig S2. The intensity pattern is shown in the inset diagrams.

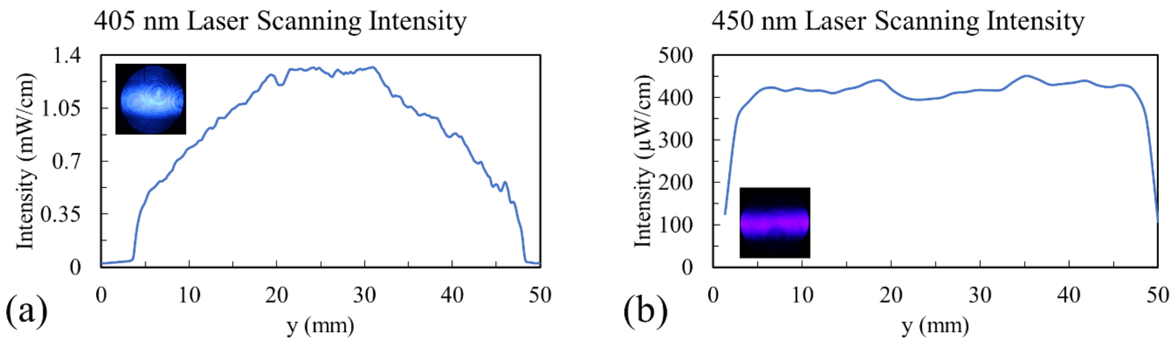

Figure S2 Transvers intensity distribution of (a) 405 nm and (b) 450 nm laser diodes.

For this work, the transfer speed of the roll-to-roll system is adjusted from 1.6 to 5 mm/sec and accompanied with multiple times exposure to achieve the suitable exposure dose at the central region of the sample. The dose can be calculated by:

$$D(y) = N\frac{l}{v} \times I(y) = N\frac{l}{v}\int_0^l I(x,y)\,dx$$

where $D(y)$ is the dose distribution along the y direction, l is the length of the illumination area along y-axis, v is the scanning velocity, N is the number of scanning times, $I(y)$ is the 1D transverse intensity profile, and $I(x,y)$ is the 2D intensity distribution.

For 405 nm wavelength scanning lithography, the nominal exposure dose of the range from 25 to 42 mJ/cm^2 yields the best patterning results. In the case of 450 nm

wavelength scanning exposure, the exposure dose varies from 140 to 160 mJ/cm^2 for the lithography process.

Supplementary Section B: Image Analysis Algorithm

To quantitatively analyze the assembly and patterning yield, we use ImageJ to examine the top-view SEM images. The phase elements used for the Talbot effect are nanospheres, therefore, both the colloidal phase mask and the resulting pattern will resemble an array of circles from the top view. The difference is that the nanospheres will yield intensity maxima at the center in the SEM signal, while the exposed photoresist consists of an array of holes and will yield intensity minima. The pattern quality is quantified by two steps: first, the circle center is identified; second, the distance between each center pair is measured to determine if they are close-packed.

In this work, a macro in ImageJ is used to automatically perform a set of filters to convert a feature in the analyzed images, as illustrated in Fig S3. This process converts a circle of a known pixel size into a dark spot around the center of this feature. The position of the center can be recognized by finding the local brightness maxima position of the resulting bright spots image. First of all, the original image is processed to identify the feature edge. A circle kernel with the expected diameter is then introduced and convolved with the feature edge image.[1] If a pixel is located near the center of the feature, the kernel will match the edge perfectly and the convolution will reach a local maximum. The convolution result can be illustrated as a grayscale image, as shown in Fig S3(c). The position and coordinates of the circle centers can then be identified using the "Find Maxima" function in ImageJ. An example of the location result is shown in Fig S3(d).

After finding the center positions, a macro is used to calculate the distance between two neighboring centers. Since the nanosphere diameter is known, this can determine if the spheres are in contact. As demonstrated in Fig S3(e), the neighborhood number of

individual nanospheres can be recorded to identify the number of close-packed neighbors. A histogram can be used to count the number of neighbors from 1-6 to determine the degree of close-packed assembly, as shown in Fig S3(f).

Most defects as point vacancy, dislocation and aggregation can be easily detected by the number of neighbors, however, the multiple layers form by the overlapping will still be identified as close-packed as showed in Fig S3(g). This kind of defect is usually from the excessive injection of the nanospheres. Under steady-state coating conditions when the nanospheres injection rate is equal to the coating rate, the close-packing monolayer forms on the liquid surface without discontinuity or overlapping. However, if the injection rate exceeds the coating rate, or in the event of environmental disturbance, the nanospheres monolayer can overlap and form multilayers. These multiple layers regions are less flexible and can form cracks upon drying on the substrate. In this case, the multiple layer area can be detected normally as monolayer regions. Yet, this defect is usually accompanied with a lot of non-close-packed nanospheres spread among the monolayer and come out with low assembly yield.

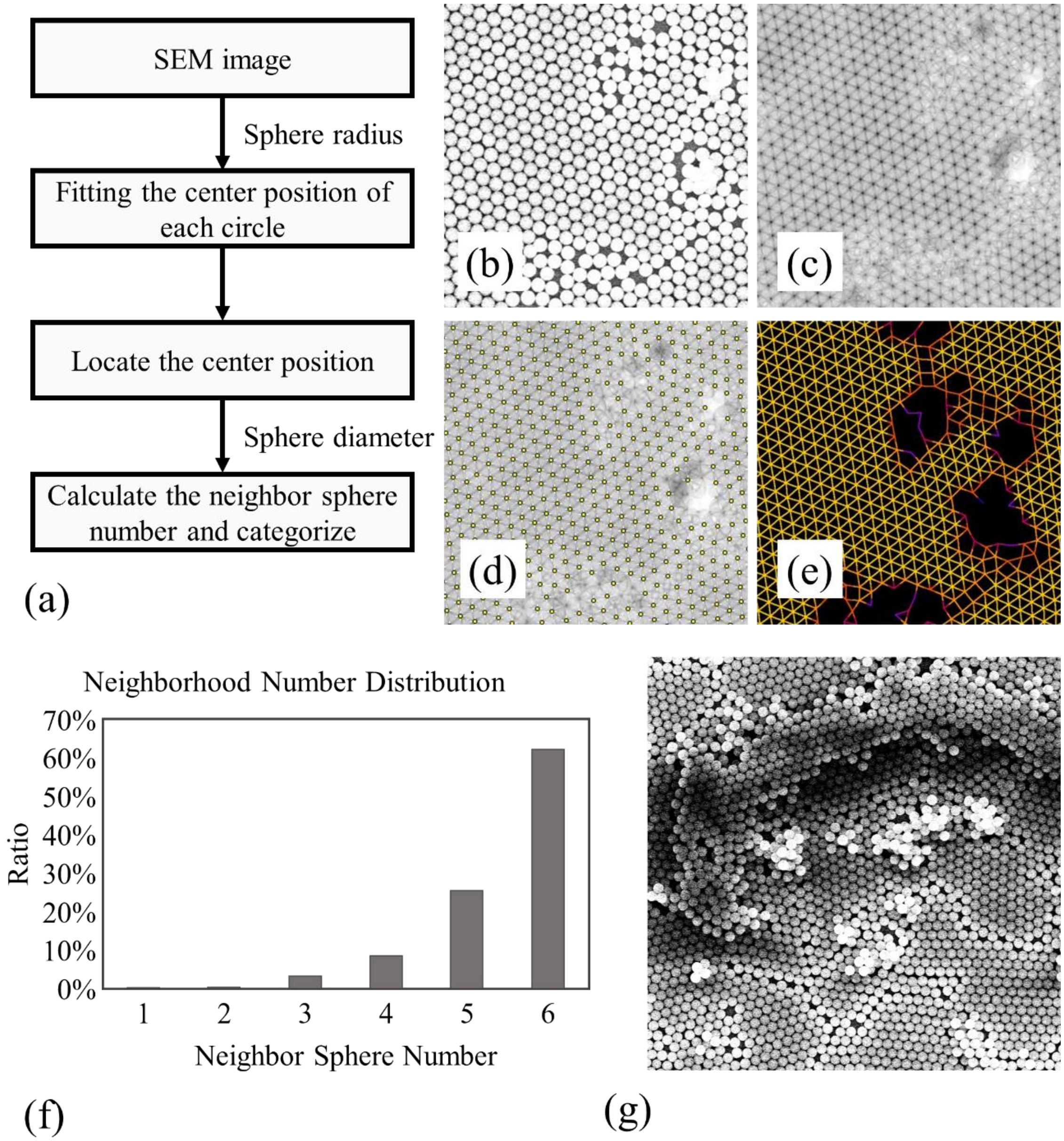

Figure S3 Image analysis algorithm to determine number of close-packed neighbors. (a) The algorithm flow chart. (b) Original top-view SEM image, (c) center fitting, (d) center locating and (e) neighborhood number determination, (f) the neighborhood number distribution, (g) the top-view SEM image of multiple layers region.

Supplementary Section C: Suspension Preparation and Concentration Comparison

Suspension with a nanosphere volume fraction range of 0.9% to 2.6% has been quantitatively examined in this work. The suspension is made by mixing silica nanospheres dry powder with butanol to desired ratio, and then sonicated to ensure the nanospheres are well-dispersed in the solvent. The sonication time varies for different concentration and ranges from 4 to 6 hours. The suspension with different concentration also results in different assembly packing result. The analysis result shows that isolated packing is the major defect of the samples made by low concentration suspension. As shown in Fig S4(a), the η_5 (fraction of 5 and 6 neighbors) decrease by about 10% if the suspension concentration is lower than 1.3%, illustrating that the grain size decreases with the concentration. Moreover, the low-packing (nanospheres with less than 5 neighbors) ratio increases from 12.8% to 20.5% when the concentration is lower than 1.3%. This also indicates that the low concentration results in a lot of vacancy, as shown in Fig S4(b).

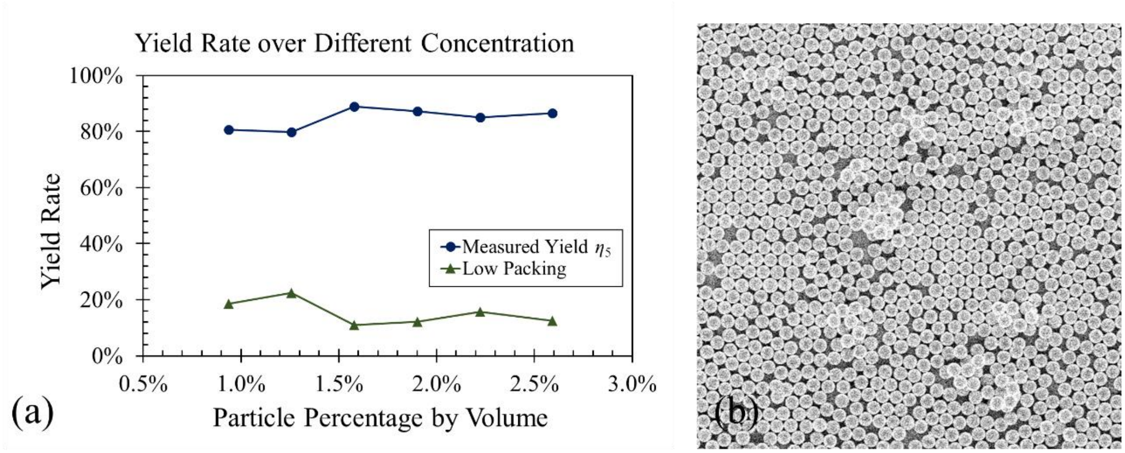

Figure S4 Assembly yield rate and SEM. (a) The η_5 and low-packing (neighbor number under 4) ratio over different suspension concentration. (b) Top-view SEM of 0.9% volume fraction suspension coating.

Supplementary Section D: Calculation of 2D yield Contour

The yield η_5 (percentage of 5 and 6 neighbors) and η_6 (percentage of 6 neighbors) of the patterned substrates can be calculated by the analysis result of an individual image for a specific position. However, to examine the yield rate distribution over a large area, an array of images array has to be obtained. In this work, an electron-beam lithography system (Raith 150 II, Raith) is employed to take SEM image array automatically. Using automated image, the path of the sample stage and the desired imaging position are pre-defined. For example, in the case of examining the colloidal particle assembly yield rate on a 100 mm silicon wafer, a 400-point coordinate array has been defined in which each imaging location is 3.5 mm apart in both horizontal and vertical directions to its next neighbor. This results 400 top-view images in a 20 by 20 matrix over a 70 by 70 mm^2 area. The system then will capture an image from each position by the predetermined imaging parameter. Prior to imaging process, the sample has to be leveled by triple-point leveling method to maintain the same focal distance over all the positions. The 400 resulting SEM images will be analyzed by the algorithm in Supplementary Section B. The statistics data, η_5 and η_6, of every SEM image will be displayed in different color as a single pixel at its corresponding location to generate a 2D yield contour, as shown as Fig S5.

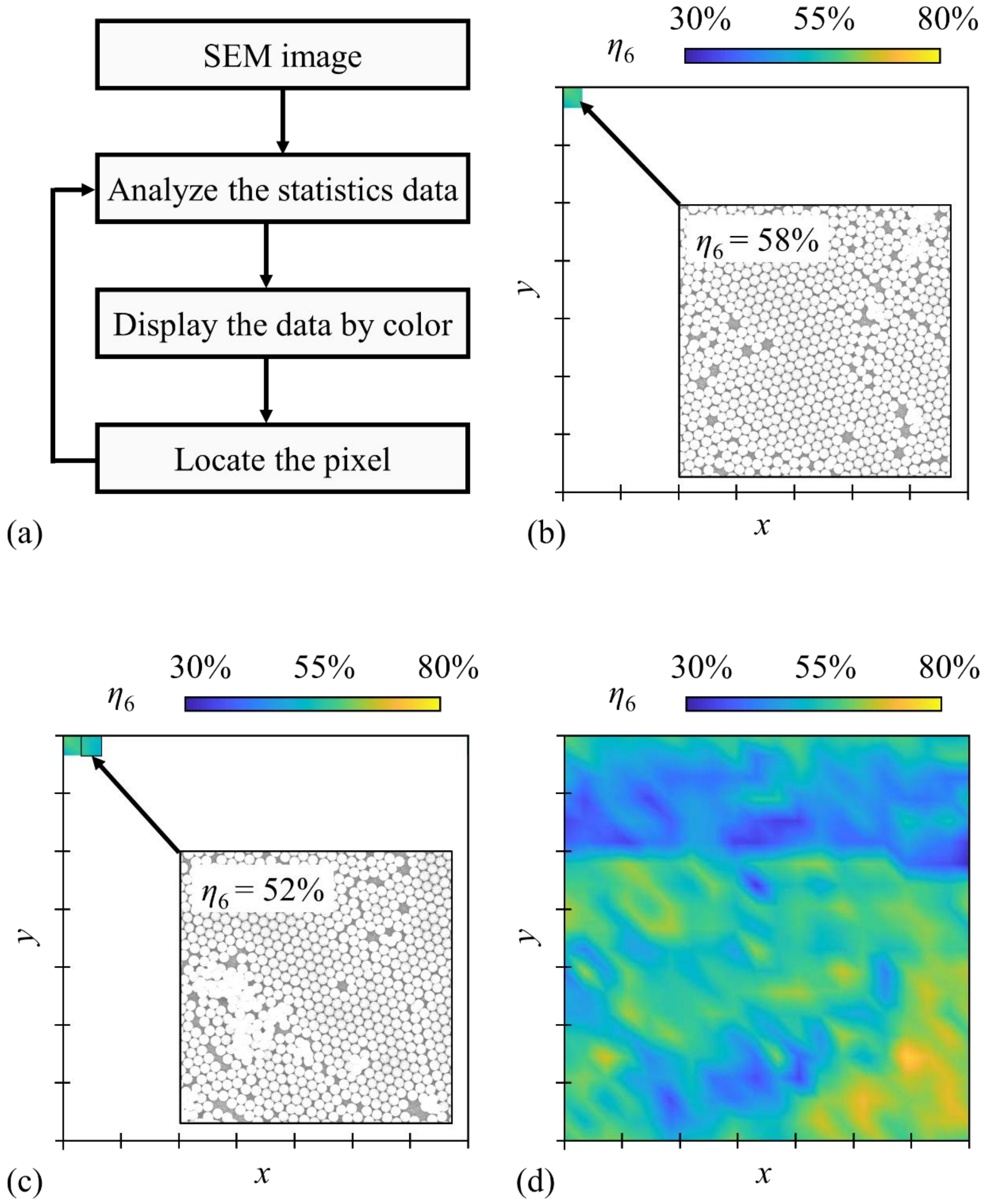

Figure S5. Yield rate 2D contour process. (a) Algorithm flow chart of 2D contour process. (b) and (c) Contour processing of each SEM image analysis result. (d) Final 2D contour of 400 SEM images.

Supplementary Section E: Scanning Exposure Dose Estimation

The intensity variation of the scanning exposure module depends on the illumination profile, therefore the dose identification after lithography process becomes more critical. According to the near-field focusing effect of the nanosphere,[2–4] the high intensity distribution patterns a hole feature on the positive photoresist right below the nanospheres. Fig S6 shows the dose test result of 500 nm nanospheres under 405 nm wavelength exposure.

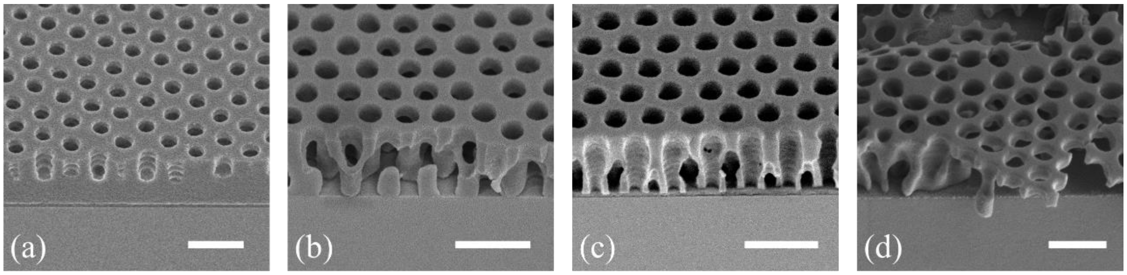

Figure S6 Cross-section SEM iamges of 500 nm period 3D nanostructure made by 405 nm wavelength scanning lithography process. The doses are (a) 25 mJ/cm^2, (b) 35 mJ/cm^2, (c) 45 mJ/cm^2, and (d) 55 mJ/cm^2.

The average holes diameter is 240 nm under 25 mJ/cm^2 exposure and the SEM cross-section image indicate only the surface layer is exposed, as shown in Fig SE 1(a). The 3D nanostructure under more suitable dose of 35 mJ/cm^2 and 45 mJ/cm^2 have hole diameters of 285 and 309 nm, as illustrated in Fig S7(b) and (c) respectively. It is notable that the 45 mJ/cm^2 case shows clear 3D nanostructures, but the side walls are relatively thin (~40 nm) under the higher exposure. Fig S6(d) shows the result of overexposure, where the side walls of the nanostructures are fully exposed that only the top layer survive. Here the average hole diameter is 333 nm.

It is interesting to observe that the hole diameter correlates well with the degree of exposure within the 3D nanostructure. This was also observed in the scanning lithography result discussed in the main manuscript. Both of these results are compared in Fig S7, where the average hole diameters are plotted versus the exposure dose. The dose of the scanning exposure is estimated base on the intensity profile, where the five different sampling point are 0, 5, 10, 15, and 20 mm from the centerline, respectively. This result indicates that the degree of exposure in 3D can be estimated by analyzing the diameter of the top 2D layer, allowing volumetric data to be extracted from surface measurements.

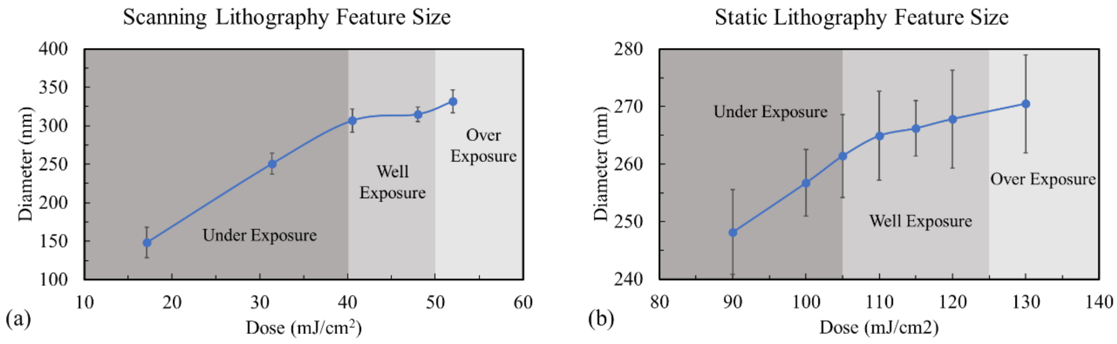

Figure S7 Top-view hole diameter of (a) scanning lithography by using 500 nm nanospheres and 405 nm laser diode, and (b) static lithography by using 500 nm nanospheres and 325 nm laser.

References

(1) Wheeler, R. RichardWheeler.net https://www.richardwheeler.net/contentpages/index.php (accessed Jul 29, 2019).

(2) Chang, C.-H.; Tian, L.; Hesse, W. R.; Gao, H.; Choi, H. J.; Kim, J.-G.; Siddiqui, M.; Barbastathis, G. From Two-Dimensional Colloidal Self-Assembly to Three-Dimensional Nanolithography. *Nano Lett.* **2011**, *11* (6), 2533–2537.

(3) Zhang, X. A.; Elek, J.; Chang, C.-H. Three-Dimensional Nanolithography Using Light Scattering from Colloidal Particles. *ACS Nano* **2013**, *7* (7), 6212–6218.

(4) Min, J.-H.; Zhang, X. A.; Chang, C.-H. Designing Unit Cell in Three-Dimensional Periodic Nanostructures Using Colloidal Lithography. *Opt. Express, OE* **2016**, *24* (2), A276–A284.
